# Atypical Skull Base Osteomyelitis of the Clivus Mimicking a Malignant Lesion: A Case Report

**DOI:** 10.3390/diseases14040138

**Published:** 2026-04-09

**Authors:** Magdalena Stocker, Johanna Felber, Patricia Bäck

**Affiliations:** Department of Otorhinolaryngology, Head and Neck Surgery, University Hospital of Salzburg, Paracelsus Medical University Clinic (PMU), Muellner Hauptstraße 48, 5020 Salzburg, Austria; j.felber@salk.at (J.F.); p.baeck@salk.at (P.B.)

**Keywords:** central skull base osteomyelitis, skull base, nasopharynx, malignant melanoma

## Abstract

Background/Objectives: Atypical skull base osteomyelitis (ASBO) is a rare disease, typically involving the basisphenoid and basiocciput. Diagnosis consists of clinical examination, imaging methods such as PET-CT scans and MRI, microbiological testing, and possibly native tissue samples. Long-term intravenous antibiotic therapy is the treatment of choice. Methods/Case Report: We present a case of ASBO of the clivus initially suspected to be a malignant lesion due to malignant melanoma in the patient’s history. Several tissue biopsies were taken, and microbiological testing of native tissue biopsies in combination with PET-CT and MRI imaging led to the diagnosis of ASBO. The patient received long-term antibiotic therapy with meropenem and drastically improved in his overall health. Discussion and Conclusions: This case highlights the challenges encountered in the diagnosis and management of ASBO, especially with relevant possible differential diagnoses.

## 1. Introduction

Skull base osteomyelitis is a rare disease that can be divided into typical (SBO) and atypical skull base osteomyelitis (ASBO) [1]. ASBO predominantly involves the basisphenoid and basiocciput [1]. Unlike SBO, which is usually the result of advanced necrotizing external otitis (so-called otogenic), ASBO does not have an otogenic cause [2,3]. It can arise from sinus infection, especially recurring sinusitis [4], but can also stem from other regional infections of the deep face or oral cavity, and even remain idiopathic [5,6,7]. It might also occur iatrogenically after surgery of the nasopharynx or skull base [8,9]. Furthermore, patients predisposed to mucosal barrier breakdown and infections of the head and neck due to osteoradionecrosis after radiotherapy have been described as more susceptible to the development of ASBO [10].

The central skull base comprises the clivus, bordered anteriorly by the nasopharynx, posteriorly by the foramen magnum, anterolaterally by the petrous temporal bone, and posterolaterally by the jugular foramen [11].

The intracranial venous drainage system consists of several levels. It is divided into the supratentorial and infratentorial drainage systems [12]. Emissary veins, diploic veins, and other veins create a complex system that enables intracranial drainage. Most of the cerebral venous drainage ultimately collects into the transverse and sigmoid sinuses of the skull base [13]. At the skull base level, relevant anatomical structures involve the internal cerebral veins, basal veins, vein of Galen, and multiple dural venous sinuses, including the straight, cavernous, and petrosal sinuses. The cavernous sinus itself is a central structure in skull base venous drainage. It receives blood from the superior and inferior ophthalmic veins, the superficial middle cerebral vein, and the sphenoparietal sinus. Importantly, it communicates with the deep venous system via emissary veins and the basilar venous plexus [14]. The basilar plexus, located on the clivus, connects the two inferior petrosal sinuses and communicates with the internal vertebral venous plexus, providing a valveless pathway that allows bidirectional flow. Its anatomical complexity, variability, and lack of valves underpin both its physiological adaptability and its vulnerability to disease [14]. Furthermore, the skull base comprises several osseous foramina and channels that are crossed by nerves and vascular structures [15]. These can be a pathway for the spreading of inflammation, and they may cause specific symptoms such as headaches and possible cranial neuropathies [1,11]. Since an inferior extension of the lesion can involve the prevertebral region up to the fourth cervical vertebrae, cervical symptoms such as pain or restriction in movement can also occur [16]. Patients might be immunocompromised due to type 2 diabetes, immunosuppressant therapy, or other preconditions [1,17,18]. Different pathogens such as Streptococcus spp., Staphylococcus aureus, Pseudomonas aeruginosa, fungal, and/or mixed infections have been described in the literature [3,19,20]. Diagnostic steps include clinical examination, laboratory testing, imaging with MRI and/or CT scans, and histopathological and microbiological examination of tissue samples [16,21]. Therapy consists of long-term systemic antibiotics with a duration of at least 6 weeks [22,23] and possible additional therapy, such as the improvement of immunocompetence with measures such as optimization of antidiabetic therapy.

Important differential diagnoses are skull base tumors of any kind, with symptoms possibly overlapping, and imaging findings at times not clearly differentiating between the two diseases. Tumors with osteolytic and/or osteoblastic characteristics, such as meningioma, neurofibroma, chordoma, chondrosarcoma, paranasal sinus malignancies, and neuroendocrine carcinoma, as well as metastases from different primary tumors, can occur in or spread to the skull base [24,25,26,27].

Malignant melanoma accounts for 1.7% of all new malignant tumors and is one of the most common types of cancer worldwide. In 2022, over 331.000 new cases of malignant melanoma were reported globally [28]. Distant metastases of malignant melanoma typically occur in skin, subcutaneous, or lymph node sites; lungs; visceral sites; and the central nervous system [29].

## 2. Case Report

We present the case of an 81-year-old male with a suspect lesion of the nasopharynx that was initially referred to us by the dermatology department. The patient had suffered from a cutaneous melanoma metastasis of his right shoulder with an unknown primary tumor (AJCC III) in 2017. The metastasis was resected, and adjuvant therapy with a monoclonal antibody was recommended, but the patient declined. He then suffered from pulmonary metastasis (AJCC IV (T0 N1 cM1b)) that was resected via video-assisted thoracoscopic surgery (VATS) in 2020. Adjuvant therapy with a monoclonal antibody (Nivolumab) was then established but discontinued due to immune-mediated arthritis. After this, he went into remission. He furthermore suffered from type 2 diabetes, ischemic dilatative cardiomyopathy, and two-vessel coronary artery disease.

**Figure 1:**
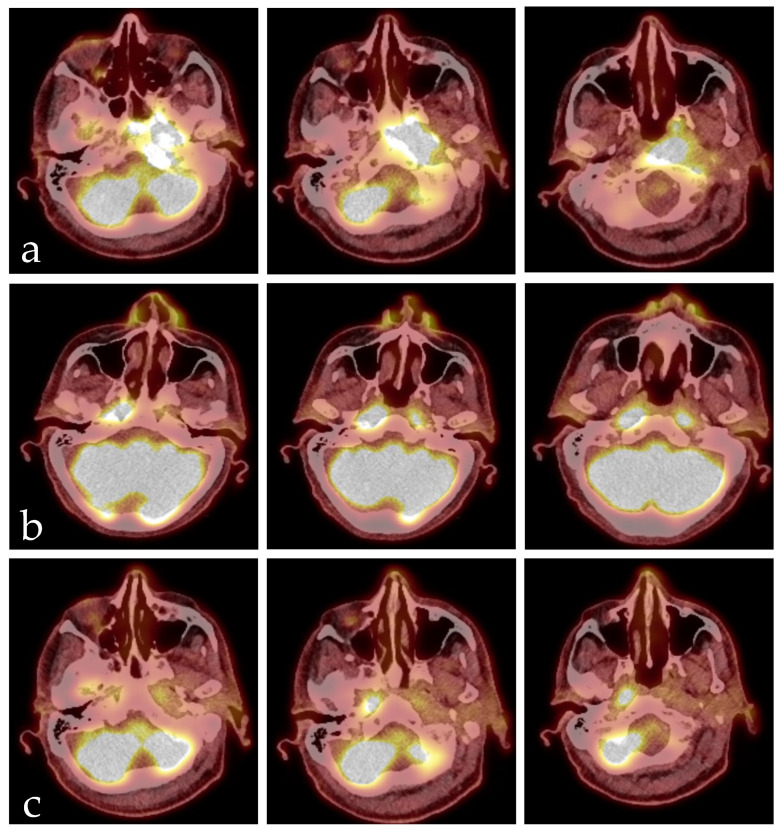
Exemplary representation of FDG-PET-CT scans the patient received from March until October 2025; CT scans are merged with PET scans. (**a**) Initial scan from March 2025 with an intense accumulation in the nasopharynx on the left side and broad-based infiltration of the skull base/clivus. (**b**) Second scan after six weeks of systemic antibiotic therapy with meropenem, showing a significant reduction in the size of the lesion in the left nasopharynx. However, a new accumulation has appeared in the right nasopharynx. (**c**) Scan two months after discontinuation of antibiotic therapy, showing only discrete residual storage visible.

He received an FDG-PET-CT scan as part of regular tumor follow-up care in March 2025, revealing a newly developed intense accumulation in the area of the nasopharynx on the left side, with broad-based infiltration of the skull base/clivus; this was highly suspicious for a secondary carcinoma, measuring 3.2 × 4.5 × 1.2 cm with a maximum SUV of 10.1 (Figure 1). The case was presented to our interdisciplinary tumor board, recommending histological confirmation in the form of a biopsy. The patient was thus referred to our ENT outpatient department in April 2025, reporting headaches, dizziness, loss of appetite, and weight loss (15 kg) in the last three months. He furthermore complained of otalgia in his left ear and was in poor general health. Endoscopy of the pharynx revealed submucosal swelling in the nasopharyngeal area. Ear microscopy showed redness of the tympanic membrane but no signs of mastoiditis. He had a mildly elevated leucocyte count of 10.3 G/L and CRP of 1.2 mg/dL. An MRI scan revealed an extensive malignant-suspicious infiltrative–destructive mass of the skull base/nasopharynx on the left, crossing the midline to the right with protrusion into the left-sided premedullary cistern and involvement of the medulla oblongata, as well as perifocal infiltration of the dura, infiltration of the left cavernous sinus, partial sinus vein thrombosis involving the left sigmoid sinus, ossary involvement and extensive perivertebral contrast enhancement caudally until C2. The mastoid region was unaffected. We performed navigated endoscopic transnasal surgery with several biopsies and initiated adequate anticoagulation due to thrombosis of the sigmoid sinus. Pathology showed an increased resorptive–histiocytic/chronic granulocytic inflammatory reaction and florid inflammatory reaction with reactive epithelial changes and gland formation with no evidence of malignancy. Since the imaging morphology strongly suggested a malignant process, we performed two further biopsies—the last one in collaboration with the neurosurgery department. No malignancy could be detected in any of the tissue samples. The patient was discharged from the hospital but had to be readmitted shortly after due to a decline in his overall health, suffering from increasing headaches, dizziness, and dehydration. We started intravenous fluids and transdermal fentanyl therapy.

Native tissue samples taken during the last surgery and tissue swabs were also sent for microbiological testing. We received the results of these samples two weeks after the last surgery, at the time the patient was readmitted. They showed evidence of Pseudomonas aeruginosa; thus, in consultation with our infectiologists, we started antimicrobial therapy with meropenem in accordance with the antibiogram, given the patient’s penicillin allergy. A native tissue examination for possible mycosis showed no evidence. Further laboratory chemistry showed elevated cANCA values. A rheumatological consultation revealed no evidence of vasculitis, but rather a suspicion of ASBO. With no malignancy in the tissue samples, no rheumatological suspicion of vasculitis, and evidence of Pseudomonas aeruginosa in the tissue samples, our working diagnosis remained ASBO, and malignancy was finally ruled out.

**Figure 2:**
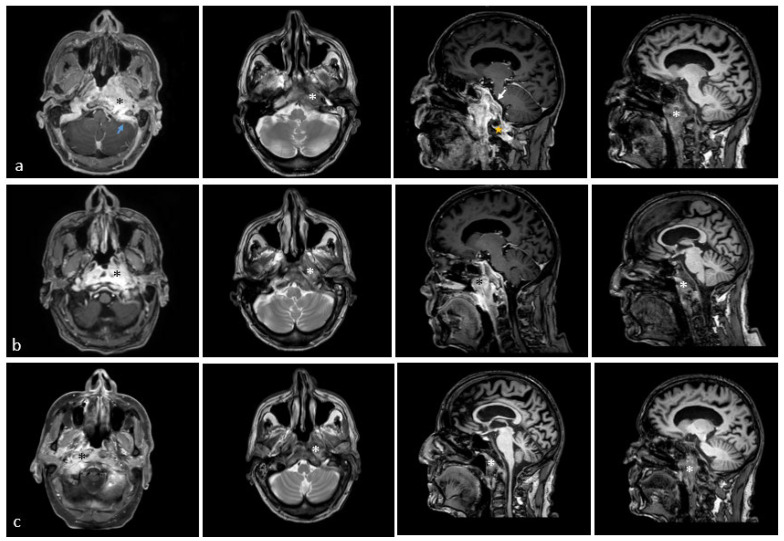
Exemplary representation of the MRI scans the patient received from April until June 2025. Axial images are represented on the left, sagittal images on the right. The first column of axial images consists of contrast-enhanced maximum intensity projection (MIP), the second column of axial images consists of native T2 images, the first column of sagittal images of b and c are contrast-enhanced compressed sensing T1 images, the sagittal image of c is a native compressed sensing T1 image, and the second column of sagittal images are native T1 images. (**a**) MRI scan from 9 April 2025 showing an extensive, malignant-suspicious mass lesion in the skull base/nasopharynx on the left side (black and white asterisk). Furthermore, broad-based involvement of the dura (white arrow) and ossary involvement/partial destruction and extensive perivertebral contrast accumulation caudally up to C2 (yellow star) are described, as well as partial sinus vein thrombosis affecting the left sigmoid sinus (blue arrow). (**b**) MRI scan from 30 April 2025 showing the known lesion with slightly regressive contrast enhancement but unchanged extent of the lesion (black and white asterisk). (**c**) MRI scan from 26 June 2025 showing the known lesion in the left nasopharynx, tending to decrease in intensity but with slightly increasing signal on the right side (black and white asterisk).

A follow-up MRI scan two weeks after initiation of antibiotic therapy revealed a decrease in the size of the osteomyelitic lesion but an increasingly hyperintense representation of the cervical soft tissues on the left paramedian and dorsal to the atlas. Consecutive MRI scans are depicted in Figure 2.

The general condition of the patient increased over the first two weeks of antibiotic therapy. We transferred him to the geriatric ward to continue antibiotic therapy. He received extensive physio-therapeutic and psychosomatic support, resulting in significantly increased overall health.

After six weeks of antibiotic therapy, we performed another FDG-PET-CT scan. It showed a significant reduction in the size of the lesion in the left nasopharynx, now measuring only approximately 0.9 cm in diameter with a maximum SUV of 9.2. However, a new accumulation had appeared in the right nasopharynx. Since the patient has drastically improved in his overall health, we assumed that imaging could lag behind clinical progress and decided to discontinue antibiotic therapy in accordance with our infectiologists. The patient was discharged from the hospital. Another FDG-PET-CT scan was performed two months after discontinuation of antibiotic therapy, showing only discrete residual storage visible with a diameter of approximately 0.5 cm and a maximum SUV of 3.8 in projection onto the left nasopharynx and multiple similarly regressed deposits. Consecutive FDG-PET-CT scans are demonstrated in Figure 1.

The patient reported no more headaches and better general condition; the only remaining symptom was dizziness. To date, there are no clinical signs of recurring osteomyelitis. The patient will receive regular MRI scans every six months as follow-up care.

## 3. Discussion

SBO, in general, and ASBO in particular, are rare clinical entities that require a thorough clinical workup and consistent long-term antimicrobial therapy [1,16]. Diagnosis of ASBO can be difficult and delayed, as clinical symptoms can be non-specific. Symptoms such as headaches, dizziness, and a decline in overall health can be associated with a multitude of diseases, including inflammatory and malignant ones, not always allowing a clear differentiation, as happened in this reported case. Cranial nerve palsies can occur in patients with ASBO, but do not necessarily facilitate differentiation between the different disease entities. Laboratory findings might also be non-specific, as elevated inflammation parameters can occur in inflammation, as well as in malignancy [2]. Radiological diagnostic tools include CT, MRI, and PET-CT scans. As Álvarez Jañez et al. state, CT is the best option for evaluating bone erosion and demineralization, MRI can help to delineate the anatomic location and extent of disease, and nuclear imaging is useful for confirming bone infection with high sensitivity [15]. However, radiological imaging of any kind might not sufficiently differentiate between inflammatory and malignant changes, as in this study [2]. Urbančič et al. discussed neoplastic disease as a principal differential diagnosis for ASBO from the radiological viewpoint [2]. An important radiological differentiator between ASBO and malignancy might lie in diffusion-weighted imaging [30,31]. In this reported case, malignancy was the initial main distractor from the correct diagnosis of ASBO, since PET-CT and MRI scans strongly suggested malignancy, and the patient had suffered from malignant melanoma in the past. This underlines the importance of considering differential diagnoses from the very beginning of the diagnostic path for non-specific clinical manifestations, as can occur in ASBO. However, it has to be noted that MRI and CT findings often lag behind clinical improvement [2,32]. This was also observed in the patient case presented here. In particular, the PET-CT images showed lesions progressing over time in some areas. However, the patient’s clinical condition had already significantly improved at the time, which led to a de-escalation of antibiotic therapy, and the patient further improved in his overall health over time.

Making matters even more difficult, biopsies may show only non-specific inflammatory changes [1,18,33,34]. This can further delay a correct diagnosis.

A clear distinction between SBO and ASBO might not always be possible, since patients might have an occult or insufficiently treated infection before the diagnosis of SBO. Furthermore, infection of the temporal bone can spread medially to the central skull base and vice versa, making the true origin uncertain in some cases [35,36].

ASBO should always be considered in patients presenting with symptoms such as headaches, cranial neuropathies, and correlated findings in imaging, especially in immunocompromised patients. Nonetheless, tissue biopsies should be included in the diagnostic workup for patients, firstly to exclude possible malignancy and secondly to obtain native tissue samples for a microbiological workup.

The diagnosis of ASBO can only be made with certainty and in a timely manner by considering all relevant findings (clinical presentation, imaging, and microbiological testing) and considering the most important differential diagnoses. This is crucial for a prompt initiation of treatment and, consequently, a higher chance of a favorable outcome for the patient.

## 4. Conclusions

We present a case of ASBO, initially assumed to be a malignant lesion due to the patient’s history of malignant melanoma and suspicious findings during imaging. Several tissue biopsies did not show any signs of malignancy, but native tissue samples showed evidence of Pseudomonas aeruginosa, leading to the diagnosis of ASBO. Systemic antibiotic therapy over several weeks led to clinical improvement and decreased signs of osteomyelitis in imaging controls. To the best of our knowledge, this is the first case report of ASBO mimicking malignant melanoma. The first pathological signs were shown in a routine PET-CT that was performed as part of a regular tumor follow-up. This case highlights two important aspects: malignant melanoma is a clinical chameleon and can present in various anatomical locations, whether it be as a primary tumor or metastasis. The second aspect is the difficulty in diagnosing ASBO.

Pitfalls in the diagnosis of ASBO are non-specific clinical symptoms and laboratory results, difficulties in radiological differentiation between inflammatory and malignant disease, and non-specific inflammatory changes in tissue samples. Nonetheless, these diagnostic tools are ultimately essential to reach the correct diagnosis.

ASBO should always be considered as a possible differential diagnosis for immunocompromised patients with diffuse clinical symptoms such as headaches and cranial neuropathies, accompanied by suspect imaging findings. Tissue biopsy should be performed if possible to rule out malignancy and strengthen the diagnosis via microbiological testing of tissue samples and swabs.

## Data Availability

Data will be made available upon reasonable request, with the prerequisite of ensuring the privacy of the patient.

## Abbreviations

The following abbreviations are used in this manuscript:

AJCC	American Joint Committee on Cancer
ASBO	atypical skull base osteomyelitis
CRP	c-reactive protein
MRI	magnetic resonance imaging
FDG-PET-CT	fluorodeoxyglucose positron emission tomography with computer tomography
SBO	skull base osteomyelitis
